# Optical Second Harmonic Generation on LaAlO_3_/SrTiO_3_ Interfaces: A Review

**DOI:** 10.3390/ma16124337

**Published:** 2023-06-12

**Authors:** Andrea Rubano, Domenico Paparo

**Affiliations:** 1 Physics Department “E. Pancini”, University Federico II, Monte S. Angelo, Via Cintia, 80126 Naples, Italy; 2 Institute of Applied Sciences and Intelligent Systems (ISASI), Consiglio Nazionale delle Ricerche (CNR), Via Campi Flegrei 34, 80078 Pozzuoli, Italy; domenico.paparo@cnr.it

**Keywords:** non-linear optics, perovskite hetero-structures, 2D electron gas

## Abstract

As we approach the limits of semiconductor technology, the development of new materials and technologies for the new era in electronics is compelling. Among others, perovskite oxide hetero-structures are anticipated to be the best candidates. As in the case of semiconductors, the interface between two given materials can have, and often has, very different properties, compared to the corresponding bulk compounds. Perovskite oxides show spectacular interfacial properties due to the the rearrangement of charges, spins, orbitals and the lattice structure itself, at the interface. Lanthanum aluminate and Strontium titanate hetero-structures (LaAlO${}_{3}$/SrTiO${}_{3}$) can be regarded as a prototype of this wider class of interfaces. Both bulk compounds are plain and (relatively) simple wide-bandgap insulators. Despite this, a conductive two-dimensional electron gas (2DEG) is formed right at the interface when a LaAlO${}_{3}$ thickness of n≥4 unit cells is deposited on a SrTiO${}_{3}$ substrate. The 2DEG is quite thin, being confined in only one or at least very few mono-layers at the interface, on the SrTiO${}_{3}$ side. A very intense and long-lasting study was triggered by this surprising discovery. Many questions regarding the origin and characteristics of the two-dimensional electron gas have been (partially) addressed, others are still open. In particular, this includes the interfacial electronic band structure, the transverse plane spatial homogeneity of the samples and the ultrafast dynamics of the confined carriers. Among a very long list of experimental techniques which have been exploited to study these types of interfaces (ARPES, XPS, AFM, PFM, …and many others), optical Second Harmonic Generation (SHG) was found to be suitable for investigating these types of buried interfaces, thanks to its extreme and selective interface-only sensitivity. The SHG technique has made its contribution to the research in this field in a variety of different and important aspects. In this work we will give a bird’s eye view of the currently available research on this topic and try to sketch out its future perspectives.

## 1. Introduction

Nowadays, almost every modern electronic device, such as computers, music players, mobile phones, as well as opto-electronic devices [1], is equipped with chip-sets made of semiconductors. However, the bulk characteristics of most semiconducting materials are rather simple. Their true advantage and their functionality lie within the transport of charge carriers across or along the interfaces between different materials. Prominent examples are p–n junctions, Schottky contacts and diodes, as well as various transistor designs [2]. Around the year 2000, the progress in the field of semiconductor technology was described by Moore’s law, which holds that the number of transistors that can be placed on an inexpensive integrated circuit doubles approximately every two years. For years, the validity of Moore’s law was supported by refined chip-set fabrications processes that allowed for rapidly shrinking transistor sizes. However, around the year 2010, the trend started to break with indications that the doubling in transistor capabilities would occur every three years instead. Approaching the limits of semiconductor technology, the search for new materials and new interface phenomena is inevitable. A promising candidate are so-called complex oxide hetero-structures. This material class was found to show spectacular interfacial properties that are not present in the majority of the constituents due to the rearrangement of charges, spins, orbitals and the re-balancing of the lattice structure at the interface. Notorious examples are BaTiO${}_{3}$/SrTiO${}_{3}$/CaTiO${}_{3}$ hetero-structures that show an enhancement of polarization compared to pure BaTiO${}_{3}$, caused by the breaking of the inversion symmetry in the hetero-structure [3]; non-superconducting BaCuO${}_{2}$ and SrCuO${}_{2}$ that show superconductivity when grown as a hetero-structure [4]; and antiferromagnetic CaMnO${}_{3}$ and paramagnetic CaRuO${}_{3}$, which show ferromagnetism at the interface [5].

In 2004, A. Ohtomo and H.Y. Hwang at Bell Laboratories discovered that, at the interface between LaAlO${}_{3}$ and SrTiO${}_{3}$, two textbook band insulators, a conducting two-dimensional electron gas (2DEG) emerges depending on the thickness of LaAlO${}_{3}$ and the precise atomic stacking sequence [6]. This discovery unleashed an intense research effort in order to understand this unexpected new property that possesses huge potential for future applications [7,8,9,10,11,12,13,14]. Most recently various new phenomena in LaAlO${}_{3}$/SrTiO${}_{3}$ interfaces were reported, ranging from superconductivity [15] and ferromagnetism [16] to tip-induced conductance [17]. In particular, the coexistence of both superconductivity and ferromagnetism, which are normally two mutually exclusive phenomena as ferromagnetism destroys the singlet correlations responsible for the pairing interaction leading to superconductivity, astonished the scientific community [18,19,20].

The large variety of properties highlights the key role of the LaAlO${}_{3}$/SrTiO${}_{3}$ interface as a model compound to understand the new physics at complex oxide interfaces. The coexistence of phases confined to a two-dimensional layer highlights the importance of a precise understanding of the LaAlO${}_{3}$/SrTiO${}_{3}$ interface on the route towards future applications. However, only little is known about the exact mechanisms that lead to the formation of the observed interfacial electronic properties. Widely-accepted models accurately describe the emergence of the conducting two-dimensional electron gas, but do not give a complete picture of the underlying phenomena and microscopic physics. In particular, this includes the localization of charge carriers [21,22]; growth-induced defects, such as oxygen vacancies [23]; and the stabilization of local inhomogeneities [17] at the interface. Hence, the question whether an extrinsic or intrinsic mechanism yields the conducting state at the LaAlO${}_{3}$/SrTiO${}_{3}$ is still a highly debated topic in the literature [24,25,26,27,28,29]. To clarify this key question, detailed knowledge about the electronic structure at the interface of the pathway to the formation of the two-dimensional electron gas is required.

**Figure 1:**
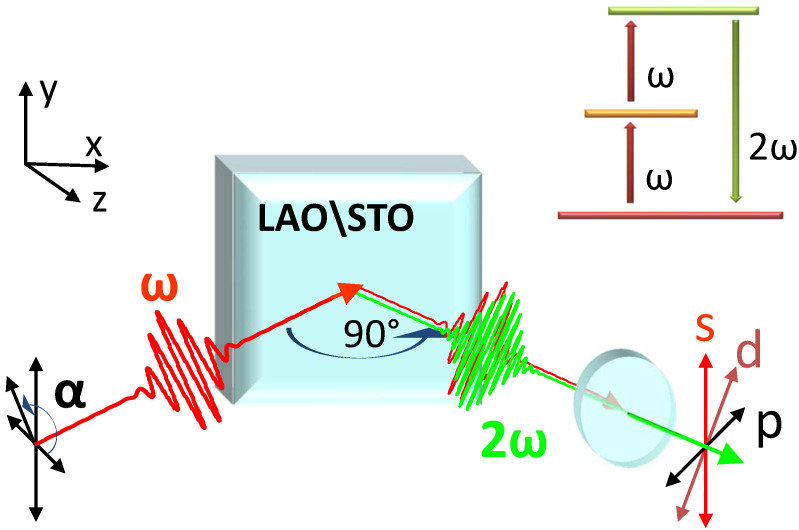
Experimental scheme and polarization geometry. The input fundamental light with frequency ω is converted to its Second Harmonic by 90${}^{\circ }$ reflection on the sample surface. The generic input polarization angle is α while the output polarization angles are fixed to be parallel to the incident plane (*p*), to the sample surface (*s*), or at 45${}^{\circ }$ (*d*). In the upper panel, the SHG energy levels diagram is shown.

The optical Second Harmonic Generation (SHG) process is essentially the generation of a light wave with the frequency 2ω by doubling the frequency ω of the incident wave [30,31,32]. The measured SHG signal, i.e., the intensity of the double-frequency wave, is proportional to the absolute square of the second order susceptibility tensor ${\hat{\chi }}^{\left(2\right)}$. The SHG levels diagram and the basic idea of the experimental layout is given in Figure 1 together with the labeling of the axes and polarization directions. The model which describes the process will be addressed in the following section. Here, we would like to focus first on the most relevant point: because of the very thin region which is affected by the 2DEG, one needs a powerful and non-invasive technique specifically sensitive to thin interfaces, and SHG is ideal with respect to these issues. The reason for this lies in the sensitivity of SHG to the symmetry of the system. A very simple and general argument shows that, under the dipole approximation, the SHG signal vanishes in any centro-symmetric medium, i.e., when the system is insensitive upon inverting each ion position from the point $\vec{r}$ to the point $-\vec{r}$. This strong symmetry can be fully achieved, evidently, only if the material extends to an infinite size in space. The presence of a surface geometrically hampers the inversion symmetry, regardless of any property of the material, and, therefore, any interface (a surface is nothing but an interface with vacuum) acts as a source of SHG signal [33]. One can regard the issue also by looking at the alteration of the arrangement of the ions in the bulk; the breaking of the inversion symmetry in a condensed matter system corresponds directly to a shift of ionic and electronic charges, and the SHG probes the resulting polarization. It is very important to understand that, for the above mentioned reasons, there is no pre-determined “probing depth” of SHG, as there is for many other surface techniques, such as ARPES and others. The optical wavelengths typically employed in experiments are in the 100 nm to the micrometer scale, and the penetration length could be, depending on the specific material, up to meters or more. This is irrelevant as the bulk of the centro-symmetric material will be illuminated by the incident light, but it will not emit any signal. The emitting region of the SHG signal instead covers the region affected by the symmetry breaking, i.e., the region where the material differs significantly by its bulk structure.

The main objective of this review is an experimental in-depth analysis of the optical non-linearities at the LaAlO${}_{3}$/SrTiO${}_{3}$ interface as driven by the electronic structure and its modification with increasing number of LaAlO${}_{3}$ mono-layers. This is achieved by exploiting the light-polarization, spectral, spatial, and temporal degrees of freedom of SHG. Throughout this review, fundamental questions such as the role of localized carriers, the presence and influence of structural inhomogeneities, and the carrier dynamics across the interface are addressed. In particular, this includes the notorious discussion about the extrinsic or intrinsic origin of the two-dimensional electron gas at the LaAlO${}_{3}$/SrTiO${}_{3}$ interface. Hence, the experimental results here presented enhance the understanding of the puzzling mechanisms yielding two-dimensional conduction, as well as the temporal dynamics involved, whose time scales are of fundamental importance for possible future applications, such as optical devices with ultrashort switching times. In the following, we will use the abbreviations LAO and STO for LaAlO${}_{3}$ and SrTiO${}_{3}$, respectively.

## 2. Theoretical Background

We report here, for the sake of clarity the first part of the SHG theory developed in Ref. [34], and we refer the reader to that work for further details.

Under very general circumstances, the dielectric polarization density, i.e., the dipole moment per unit volume $\vec{P}\left(t\right)$ can be expanded as the electrical field power series of the incoming wave $\vec{E}\left(t\right)$. A simplified expression for this, that terminates the Taylor expansion to the third term, is the following:

(1) $$\vec{P}\left(t\right)∼\varepsilon_{0}\left({\hat{\chi }}^{\left(1\right)}\vec{E}\left(t\right)+{\hat{\chi }}^{\left(2\right)}{\vec{E}}^{2}\left(t\right)+{\hat{\chi }}^{\left(3\right)}{\vec{E}}^{3}\left(t\right)\right)$$

where $\varepsilon_{0}≃8.85\times 10^{-12}$ F·m${}^{-1}$ is the dielectric constant in vacuum and ${\hat{\chi }}^{\left(n\right)}$ are the expansion coefficients, also known as non-linear susceptibilities. The non-linear polarization $\vec{P}\left(t\right)$ will act as a source of electromagnetic radiation in the wave equation. We are interested particularly in the second term, where the second power of a field oscillating at frequency ω will clearly give an oscillation of the non-linear polarization at double frequency 2ω, and, therefore, a far-field wave $E_{\mathrm{SHG}}$ oscillating at this frequency. Equation (1) does not show the tensorial nature of the non-linear susceptibilities ${\hat{\chi }}^{\left(n\right)}$ explicitly. Assuming the oscillatory behavior of second polarization term ${\vec{P}}^{\left(2\right)}\left(t\right)$ and the electric field $\vec{E}\left(t\right)$ with complex amplitudes P(ω) and E(ω), respectively, let us rewrite the second term alone, assuming the sum over repeated indices:

(2) $$P_{i}^{\left(2\right)}\left(2\omega \right)=\varepsilon_{0}\chi_{ijk}\left(2\omega \right)E_{j}\left(\omega \right)E_{k}\left(\omega \right)$$

where the apex (2) has been removed on the tensor components for the sake of simplicity. It is seen in Equation (Section 2) that if we apply the inversion operator on centro-symmetric crystals, all vectors (as they are true vectors and not pseudo-vectors) are changing their sign, while the material-related susceptibility does not (because of the inversion symmetry), so that we obtain $P^{\left(2\right)}\left(2\omega \right)=-P^{\left(2\right)}\left(2\omega \right)$ which can hold true only if the SHG signal is vanishing, as already discussed in the Introduction.

To be useful, the SHG technique must establish a clear interpretive link between the measurable ${\hat{\chi }}^{\left(2\right)}$ “macroscopic” tensor (or its variations) and the microscopic quantities of interest, for example in terms of electronic band occupation, orbital reconstructions, etc. Regardless, before entering this complex problem, we should first recognize that, in case of LAO/STO as well as any other interface, there are two possible sources of signal, i.e., two distinct interfaces, the LAO/STO and the LAO/air (or vacuum), and the signals from them can also interfere as the sub-wavelength distance of the two regions ensures a strong coherence in any given experimental condition. In the case of LAO/STO hetero-structures, the SHG signal, comes predominantly from the LAO/STO interface and it is generated mainly on the STO side of the interface [35]. It should be not a surprise, therefore, that a non-vanishing SHG signal was found also in the bare STO substrate, and its characteristics are essential—as we will discuss in the following sections—as a reference for evaluating the alterations induced by the presence of a LAO overlayer, instead of air.

The intensity of the SHG signal is the actually measured quantity in SHG experiments. Depending on the particular experiment, we can change the parameter which drives the changes in the SHG signal, we record SHG Spectroscopy as a function of the incident photon energy ℏω, SHG Imaging as a function of the spatial coordinates on the sample surface, SHG Polarimetry as a function of the input/output waves polarizations, and so forth, not to mention external parameters, such as temperature or bias electromagnetic fields. In every case the signal is proportional to the square of the reflected SHG electric field, $I_{\mathrm{SHG}}\propto {\left|E_{\mathrm{SHG}}\right|}^{2}$. Equation (2) refers to the electromagnetic fields inside the material, but we can only access the fields outside of it. Therefore, we need to re-write that equation keeping into account the Fresnel tensors ${\hat{L}}^{in,out}$ which couple the electric fields of the incident and emitted light to those which are present inside the interface. By also taking into account the time dependence of the fields and after a straightforward calculation, we have:

(3) $$E_{\mathrm{SHG}}=\frac{i\omega E_{0}^{2}}{\varepsilon_{0}c\cos\beta }\chi_{\mathrm{eff}}$$

where $c≃2.99\times 10^{8}$ m/s is the speed of light in vacuum, $E_{0}$ is the amplitude of the impinging wave, β is the angle of incidence, and $\chi_{\mathrm{eff}}$ is defined as follows:

(4) $$\chi_{\mathrm{eff}}=\int e_{i}^{\mathrm{out}}L_{ii}^{\mathrm{out}}\chi_{ijk}L_{jj}^{\mathrm{in}}L_{kk}^{\mathrm{in}}e_{j}^{\mathrm{in}}e_{k}^{\mathrm{in}}dz,$$

where $e_{i,j,k}$ are the optical polarization unit vectors in a vacuum. This approach is valid as long as the integration thickness is much smaller than the wavelength.

We now introduce a Cartesian reference system having the interface plane oriented along the xy plane and the *z*-axis perpendicular to it, in such a way that the incident plane is xz. We assume that both input and output waves are linearly polarized, with an arbitrary polarization angle α with respect to the incidence plane. The electric field unit vectors are then the following:

(5) $$\begin{array}{ccc} e^{\mathrm{in}} & = & \left(\cos\alpha \cos\beta ,\sin\alpha ,\cos\alpha \sin\beta \right)\\ e^{\mathrm{out}} & = & \left(-\cos\alpha \cos\beta ,\sin\alpha ,\cos\alpha \sin\beta \right) \end{array}$$

At this point it is very convenient to introduce a short notation for specific polarizations: *p* for a direction parallel to the plane of incidence (α=0), *s* parallel to the interface plane (α=π/2), and *d* oriented about 45${}^{\circ }$ between *p* and *s* (α=π/4).

As mentioned, we can neglect the contribution of the LAO–air interface, and so the three non-zero components of the Fresnel tensor L can be approximated by the following expressions

(6) $$\begin{array}{ccc} L_{xx} & = & \frac{2\cos\beta^{\prime }}{n\cos\beta +\cos\beta^{\prime }}\\ L_{yy} & = & \frac{2\cos\beta }{\cos\beta +n\cos\beta^{\prime }}\\ L_{zz} & = & \frac{2\cos\beta }{n(n\cos\beta +\cos\beta^{\prime })} \end{array}$$

where *n* is the refractive index of the SHG-active interface, the STO substrate in this case and $\beta^{\prime }$ the propagation (refraction) angle inside the medium. Let us note that the refractive index is dependent on the light frequency. Therefore, it must be calculated at ω (2ω) when considering the input (output) wave.

At room temperature, SrTiO${}_{3}$ crystallizes in the ABO${}_{3}$ cubic perovskite structure. The presence of a (001)-cut surface/interface lowers the symmetry to have a four-fold rotation around the surface normal and two vertical mirror planes, so that the space group it belongs to is the 4mm in the Hermann–Mauguin notation, or $C_{4v}$ in the Schoenflies notation, which possesses five different irreducible representations, named A${}_{1}$, A${}_{2}$, B${}_{1}$, B${}_{2}$, and E. When considering the material electronic states associated with these symmetry representations, the first four identify singlet states and the last a doublet. A${}_{1}$ is the fully symmetric state, which behaves like 1, *z* or $z^{2}$; A${}_{2}$ is odd with respect to both $\sigma_{v}$ and $\sigma_{d}$ inversion planes and behaves like the rotation matrix $R_{z}$; B${}_{1}$ is odd with respect to π/2 rotations and $\sigma_{d}$ reversals, and behaves like $x^{2}-y^{2}$; B${}_{2}$ is odd with respect to π/2 rotations and $\sigma_{v}$ reversals and behaves like xy, and the doublet E behaves like the variables *x*, *y* or the products xz, yz [36].

The allowed tensor component of the non-linear susceptibility for the 4mm group are:

(7) $$\begin{array}{cc} & \chi_{zzz}\\ & \chi_{zxx}=\chi_{zyy}\\ & \chi_{xxz}=\chi_{yyz}=\chi_{xzx}=\chi_{yzy} \end{array}$$

By substituting Equations (5)–(7) in Equation (4) we obtain the expression of $\chi_{\mathrm{eff}}$ for the noticeable polarization combinations shown in Figure 1 *p*-input *p*-output (pp), *s*-input *p*-output (sp), and *d*-input *s*-output (ds),

(8) $$\begin{array}{ccc} \chi_{sp}^{\mathrm{eff}} & = & \chi_{zxx}\delta L_{zz}^{\mathrm{out}}{\left(L_{yy}^{\mathrm{in}}\right)}^{2}\sin\beta \\ \chi_{ds}^{\mathrm{eff}} & = & \chi_{xxz}\delta L_{yy}^{\mathrm{out}}L_{yy}^{\mathrm{in}}L_{zz}^{\mathrm{in}}\sin\beta \\ \chi_{pp}^{\mathrm{eff}} & = & \chi_{zzz}\delta L_{zz}^{\mathrm{out}}{\left(L_{zz}^{\mathrm{in}}\right)}^{2}\sin^{3}\beta +\left(\chi_{zxx}dL_{zz}^{\mathrm{out}}L_{xx}^{\mathrm{in}}\right)-2\chi_{xxz}dL_{xx}^{\mathrm{out}}L_{zz}^{\mathrm{in}})L_{xx}^{\mathrm{in}}\sin\beta \cos^{2}\beta , \end{array}$$

where δ denotes the effective thickness of the polar interface and the $\chi_{ijh}$ are space-averaged across this thickness. Now the choice of the input/output polarization can be understood; only one element of ${\hat{\chi }}^{\left(2\right)}$ is present in sp and ds expression, and namely $\chi_{zxx}$ and $\chi_{xxz}$ respectively, while the third element $\chi_{zzz}$ cannot be experimentally isolated, but it can be calculated starting from experimental data after proper subtraction of the sp and ds components. The sets of sp, ds, and pp polarizations provide the full set of tensor elements; although it is not the only choice, it is the most simple and straightforward for the experiments. It is worth noting that a similar calculation has been carried out for other crystal orientations as well and, namely, (110), symmetry group mm2 in Hermann–Mauguin notation or $C_{2v}$ in the Schoenflies notation and (111), symmetry group 3m or $C_{3v}$. The explicit calculations are present in Ref. [37], together with the SHG experimental results on those particular crystal orientations, we will briefly discuss these non-standard orientations in a dedicated section of this review and we remind the reader to that reference for details.

When discussing the resonance structure of ${\hat{\chi }}^{\left(2\right)}$, one has first to make very general assumptions, including that (i) the bulk system is perfectly centro-symmetric, (ii) the surface/interface induced symmetry breaking only occurs along the *z* direction, and (iii) the effect of the symmetry breaking is not too large, i.e., it can be evaluated within the dipole approximation. These points imply that the parity is broken along *z* but not along *x* and *y*, so that the interfacial electronic states can be either even or odd by inversion of the *x*,*y* coordinates. Moreover, we recognize that the full 4mm symmetry holds only in the center of the Brillouin zone (Γ point), whereas in other points it is partially broken by the crystal momentum k≠0. Finally, we remark that any resonant SHG can be of three types: resonant at the fundamental photon energy, resonant at the doubled photon energy, and resonant at both energies. Having all this in mind, one additional ingredient is needed before discussing the SHG resonances, the material band-structure.

## 3. LAO/STO Structure and Properties

**Figure 2:**
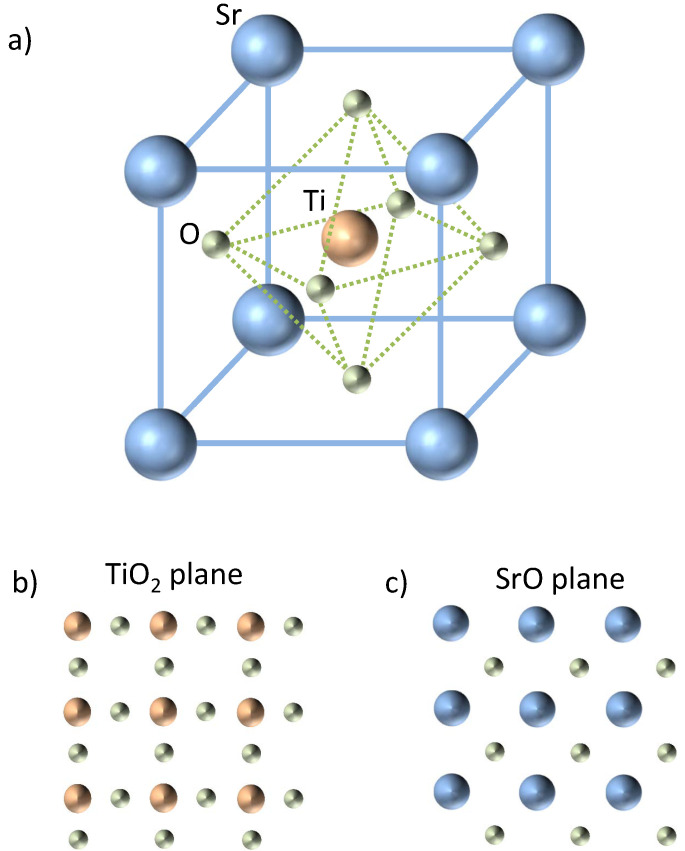
(**a**) 3D SrTiO${}_{3}$ perovskite unit cell. The solid lines represent the cubic cell borders while the dashed lines represent the TiO${}_{6}$ octahedral cage which highlights the Ti-O coordination. (**b**,**c**) 2D view of the titanium oxide (**b**) and strontium oxide (**c**) planes.

The bulk optical gap of STO is known to be associated primarily with a transition from oxygen 2*p* to titanium 3*d*-$T_{2g}$ orbitals. Its typical perovskite crystal structure is sketched in Figure 2. The Ti${}^{4+}$ ions are sixfold coordinated by O${}^{2-}$ ions, while each of the Sr${}^{2+}$ ions is surrounded by four TiO${}_{6}$ octahedra, highlighted in green in the figure. Thus, each Sr${}^{2+}$ is coordinated by 12 O${}^{2-}$ ions. Inside the TiO${}_{6}$ octahedra, while a hybridization of the O${}_{2p}$ states with the Ti${}_{3d}$ states leads to a pronounced covalent bond, Sr${}^{2+}$ and O${}^{2-}$ have an ionic bonding character. Hence, SrTiO${}_{3}$ has mixed ionic-covalent bonding properties and this leads to a unique structure, which makes it a model electronic material. For a given planar direction, for example (100), which is the most commonly used direction for growing LAO/STO hetero-structures, there are always two distinct types of equally spaced alternating atomic planes, with different arrangements of the two components, namely SrO-planes and TiO${}_{2}$-planes, sketched in Figure 2b,c. A transition from cubic to tetrahedral symmetry occurs if the temperature is lowered below approximately 100 K, or if a foreign cation/dopant is introduced into the lattice. The distortions are attributed to three main effects, dimensional effects, deviations from the ideal composition and the Jahn-Teller effect. An important example, which will be discussed in detail in the next sections, belongs to the second class. Oxygen-deficient strontium titanate is formed if the valence of the Ti cation is changed, either by heat treatment in an oxidizing/reducing atmosphere or by doping in the Sr sub-lattice. The oxygen content varies accordingly and the oxygen vacancies are sorted preferentially with respect to the local structure, i.e., a tetrahedral coordination. Non-stoichiometric thin layers of LAO can alter the ionic organization too via proximity effects, which lead to non-trivial behavior of the 2DEG at the interface. The second order Jahn–Teller effect is responsible for other types of distortions of the perovskite structure, namely a ferroelectric distortion of the cations at the Sr site, but this issue will not be addressed here.

**Figure 3:**
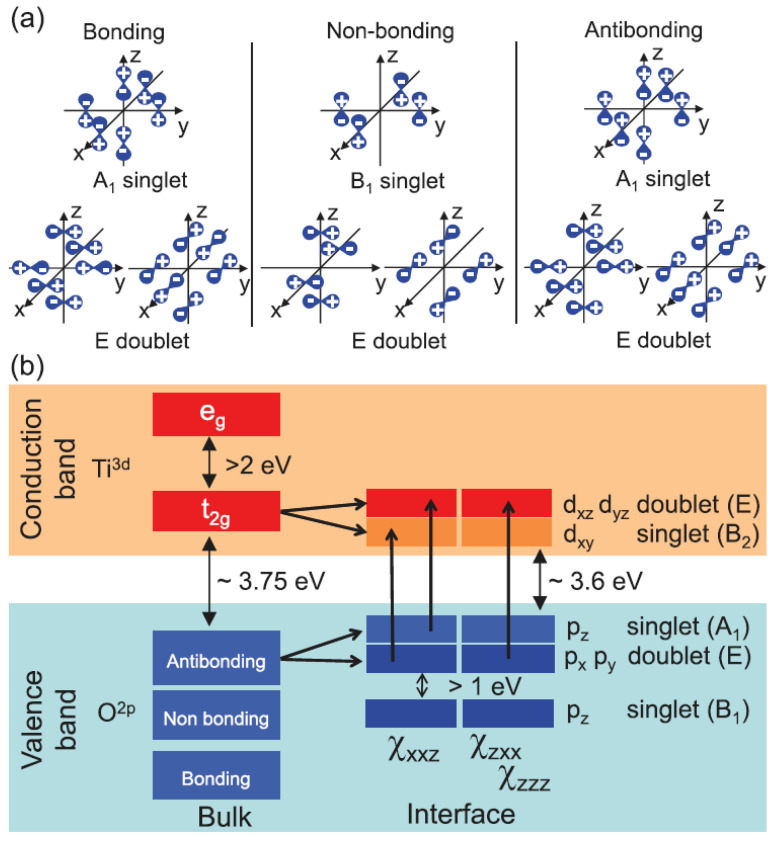
(**a**) Different arrangements of O (2*p*) orbitals forming the highest occupied molecular orbitals (HOMO) are indicated. In the bulk, they are grouped in triply degenerate levels according to the cubic m3m symmetry, but at the surface the symmetry is broken (local 4mm symmetry) and the degeneracy between the singlet and doublet states is removed. (**b**) The O(2*p*)–Ti(3*d*) 2ω transitions allowed for all three symmetry components $\chi_{ijk}$ within the symmetry group 4mm according to Equation (7). Reprinted with permission from Ref.[38].

In its stoichiometric form, SrTiO${}_{3}$ is an insulator with an indirect band gap of 3.2 eV (at T=0 K) separating the valence band and conduction band. The six-fold coordination of Ti ions from the surrounding oxygen cage creates a crystal field which splits the degenerate states of Ti−3d by 2.4 eV between $T_{2g}$ and $e_{g}$ symmetries. Figure 3a shows a sketch of the electronic structure where the cation has electron configuration $d_{0}$. The valence band which corresponds to the highest occupied molecular orbitals (HOMO) is composed mainly of oxygen orbitals 2s and 2p and the conduction band which corresponds to the lowest unoccupied molecular orbitals (LUMO) is mainly cationic, arising from empty Ti-*d* states. The gap between the HOMO and LUMO states makes SrTiO${}_{3}$ a band insulator. The Sr cations in SrTiO${}_{3}$ are, in general, strongly electro-positive and, therefore, play a minor role in the electronic structure. In any case, they play an important role in modifying the TiO${}_{6}$ connectivity of the perovskite structure and, thus, the electronic structure, but this happens mainly because of their physical size, i.e., the fraction of the unit cell volume they occupy. Indeed, the presence of “intrinsic defects”, such as ionic vacancies or structural displacements can lead to modifications of the electronic structure and electronic conductivity of the material, just in a similar way to extrinsic defects, such as dopants. Adding vacancies or doping elements in the SrTiO${}_{3}$ lattice creates defects with an effective charge relative to the host lattice. In this review, we will focus mainly on two different source of intrinsic and extrinsic effects, and namely oxygen vacancies and Nb${}^{5+}$ (in replacement of Ti${}^{4+}$ ions) dopants. The former plays a crucial role in the discussion about the origin of the 2DEG, as for many years the exact role of oxygen vacancies, which are to some extent very difficult to control, generated a long-lasting scientific dispute. The latter has been chosen as a typical source of extrinsic doping, to be compared with the intrinsic vacancy-induced conduction and with the interface-induced 2DEG.

## 4. Experimental Methods

The experimental methods used to prepare the samples and to perform the SHG measurements are different for every work covered in this review. Nonetheless, in this section we will give a very brief overview on the most common and widely used techniques and we would like to remind the interested readers to the cited references for more details. Thin LaAlO${}_{3}$ films are grown on SrTiO${}_{3}$ usually by Pulsed Lare Deposition (PLD) technique, assisted by high-energy electron diffraction (RHEED) oscillations, used to monitor the epitaxial growth and to count the number of deposited monolayers. Typical growth temperature and base oxygen pressure can significantly vary, but usual values are about ∼800 ${}^{\circ }$C and $10^{-4}$ mbar. In fewer cases, the growth technique of choice is Molecular Beam Epitaxy (MBE) with substrate temperatures in the range 700–800 ${}^{\circ }$C. Typical values of interfacial sheet conductance are $\sigma_{S}$=$10^{-5}$– $10^{-4}$$\Omega^{-1}$ at 300 K for samples above conduction threshold. The typical light-source for the experiments here reported is a Ti:Sa laser with ultrashort pulses ranging between 35 and 130 fs, with pulse energies in the range of the few μJ and fluences around 1–10 mJ/cm${}^{2}$. For spectroscopy experiments, the laser fundamental light at 800 nm is converted by an Optical Parametric Amplifier to any wavelength in the range 250–2500 nm. The optical setup depends on the specific experiment, of course, but, in general, the emerging SHG signal is collected in reflection geometry, for several reasons. First of all, the presence of a thick bulk substrate may contribute to the signal via electric quadrupole, hampering the effort of achieving an interface-only sensitive signal. On the other hand, the back side is usually not optically polished and it is covered by silver glue, which is necessary during the sample growth. Therefore, the glue should be removed after growth, with the risk of contaminating the interface side of the sample. The fundamental light is finally filtered out by optical filters, and the SHG is usually detected by means of photo-tubes or, for imaging experiments, projected with photographic objectives onto a high-sensitivity liquid-nitrogen-cooled digital CCD camera.

## 5. SHG Spectroscopy

**Figure 4:**
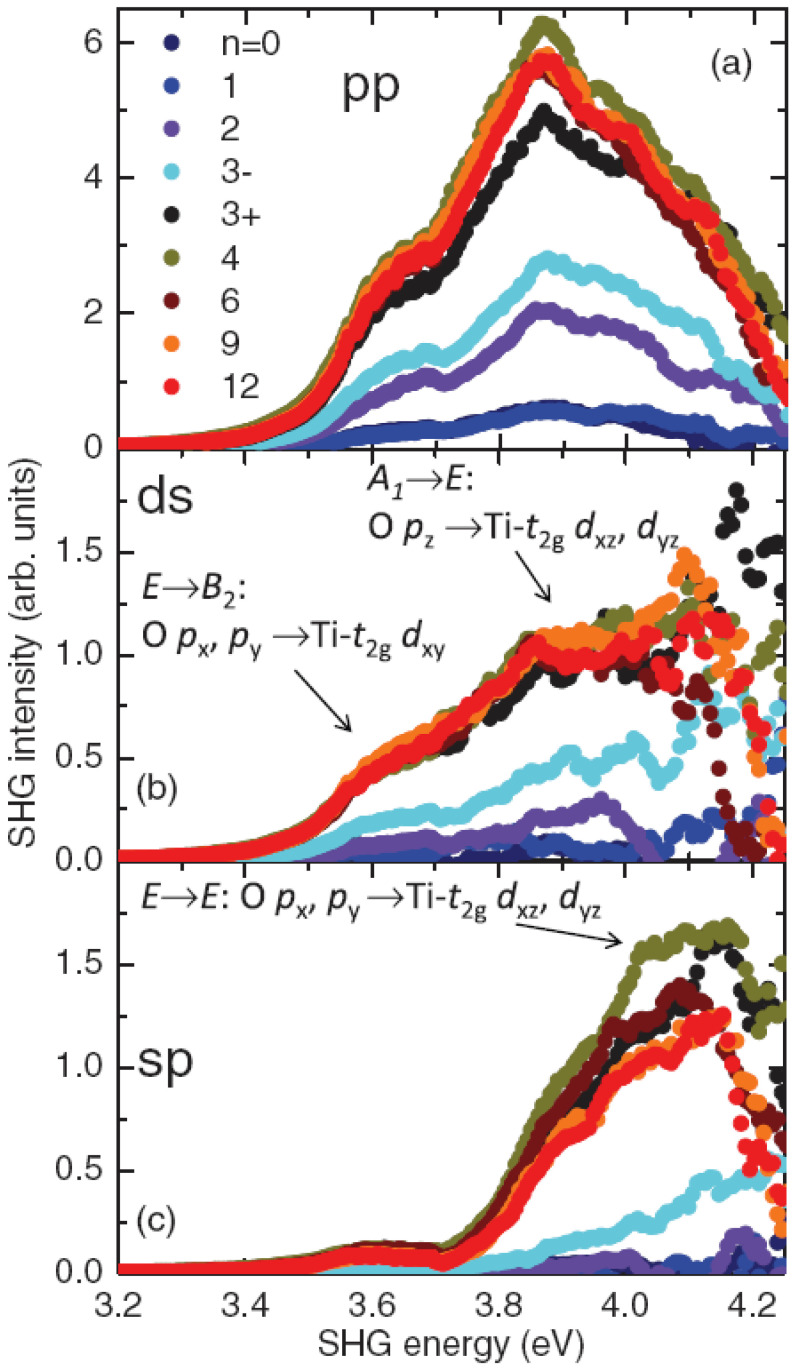
SHG spectra of LAO/STO hetero-structures for (**a**) pp, (**b**) ds, and (**c**) sp polarization configurations with different LAO thickness *n*. Samples with n=3 show strong sample-to-sample variation in SHG yield. This value of *n* represents a threshold for a discontinuous interfacial reconstruction which anticipates the conduction, i.e., the presence of free charge carriers. Here, the two extreme cases have been labeled with “+” or “−” in order to distinguish them, so that the “3+” sample is the one with the highest SHG signal, and “3−” the one with lowest SHG signal. These spectra cover the direct O(2*p*)→Ti(3*d*) band-gap transition of STO, which appears as an increase in SHG intensity at approximately 3.6 eV. The symmetry-based SHG selection rules take into account that the lowest energy transition at 3.6 eV is present in the spectrum of pp and ds, but not in sp. The same arbitrary scale is used in all three panels. Reprinted with permission from Ref. [38].

Figure 4 shows the pp, ds, and sp spectra for different LAO coverage *n* ranging from 0 to 12 mono-layers in the energy range 3.2–4.2 eV. Between 1.5 and 3.2 eV all spectra are flat and featureless and, therefore, this energy range is not very informative from a spectroscopic point of view and it is not shown in the figures. On the other hand, energies above 4.2 eV are difficult to access experimentally. A marked increase in SHG yield of about 3.6 eV is visible in all samples, and this energy corresponds to the edge of the direct O(2*p*)→Ti(3*d*) band-gap transition in STO [35,39]. However, the main peak is found at around 3.8–4.0 eV. Looking to the ellipsometry data found in the cited literature [40,41], this energy corresponds to a maximum of the dielectric function. Such a clear spectroscopic feature in the SHG spectra shows that the SHG probes the STO electronic structure and it is driven by the O(2*p*) and Ti(3*d*) orbitals involved in the local surroundings of the interface. Another important piece of information is obtained by the comparison of different SHG spectra as a function of *n*, where *n* is the number of LAO over-layers. The integrated SHG yield is small for n=0 and n=1 and it becomes slightly larger for n=2. Regardless, at n=3, a sudden and substantial boost in all components and across the spectral range takes place. Let us note that different n=3 samples can exhibit a really strong sample-to-sample variability. Let us remark here that the onset of 2D conduction at the interface happens only when n≥4 and none of the n=3 samples is found to be conductive, including those with high SHG yield. The observation of a threshold value for a discontinuous structural transition preceding the onset of conduction was first reported in 2009 by Savoia and co-authors [42] in the seminal paper “Polar catastrophe and electronic reconstructions at the interface LaAlO${}_{3}$/SrTiO${}_{3}$: Evidence from optical second harmonic generation” and has since been confirmed by other works [43]. In order to visualize the sample-to-sample fluctuations at n=3, only two samples showing maximum and minimum SHG yield are labeled, respectively, “3+” for the highest SHG signal (similar to samples with n>3) and “3−” for the lowest signal (comparable to samples with n<3). The origin of such fluctuations at n=3 is still unknown at present, but it is not surprising that, in the proximity of a phase transition, the reconstruction threshold is particularly sensitive to slight changes in the growth conditions. For a larger amount of LAO layers, the SHG signal is stable with only a minor increase with respect to the n=3+ sample, indicating that the interface is fully reconstructed. Again, no major changes of SHG are found between n=3+ and n=4, i.e., the threshold for bidimensional conduction. SHG spectra have been taken at low temperature, in the range from 10 K to 300 K, as well as with different capping materials (NdGaO${}_{3}$—NGO—and LaGaO${}_{3}$—LGO—in place of LAO) [38]. In the low-temperature regime, SHG is enhanced in all components and all samples. In particular, the band-edge 3.8 eV peak in pp and ds spectra experiences a larger enhancement than other spectral components for both STO and LAO/STO samples. More importantly, at very low temperatures (∼10 K), the number of resonances revealed by the SHG spectra exceeds the number of those identified by the SHG selection rules. This is due to the energy shifts of different sub-bands, as reported by angle resolved photoemission spectroscopy (ARPES) at similar temperatures [44]: the degeneracy of the $d_{xz}$, $d_{yz}$, $p_{x}$, and $p_{y}$ bands, is lifted and ARPES data indicate that the SHG peak splitting of about 120 meV is in good agreement with this picture. The mechanisms are still not clear, but possible candidates are the electron-confinement effect, spin–orbit coupling, and/or low-temperature tetragonal/orthorhombic distortions. Regarding the different capping layers, different topmost materials share the same qualitative behavior. This confirms that SHG is probing the electronic inter-band transitions of STO rather than any specific transition of the polar overlayer, whose effect is only visible through the changes it induces in the STO underneath. However, a few quantitative differences with respect to the specific overlayer materials can be cited, including (i) the SHG signal of the LAO/STO samples is always larger than that of the other two interfaces, (ii) in the ds and pp spectra of conductive samples the high-energy part is less enhanced in LGO/STO and NGO/STO samples than in LAO/STO, and (iii) in the pp spectra of insulating samples the overall SHG yield of LAO/STO is larger than that of the other samples.

SHG Spectroscopy has been the most important tool in order to address the problem of the microscopic origin of SHG in these materials and its relationship with the physical effect under investigation, i.e., the 2D insulator-to-metal phase transition. SHG indirectly senses the interfacial charge asymmetry. This asymmetry may be induced by a structural reorganization of the interface (in particular by Ti ions displacement) or by the presence of an electric field. In the second case, for instance, when a charged region is created close to the interface, the consequent interfacial electric field polarizes all the orbitals involved in the SHG emission and when the charge injection occurs, the SHG signal is enhanced. Therefore, the fact that the SHG signal jumps at n=3 means that charges start to be injected one layer “before” the onset of conduction, and thus they must be localized. They contribute to the interfacial field build-up, but not to transport. If the injected charges accumulated exactly at the interface, they would provide a perfect screening of the LAO polarization so that there would be no evidence of electric-field build-up in STO. On the contrary, the injected charges diffuse over a few unit cells because of kinetic energy, and create a charge gradient and, consequently, an electrostatic field that spatially decays in STO [45]. Regardless, it may seem, at a first glance, surprising that the emergence of conduction is completely separated from the orbital reconstruction. Possible solutions for this conundrum are (i) SHG is less sensitive to mobile charge than localized ones; (ii) the n=4 LAO layer promotes to conduction some of the localized carriers at n=3, so that the total carrier density does not change; and (iii) the conductive carrier density is much smaller than the localized one.

**Figure 5:**
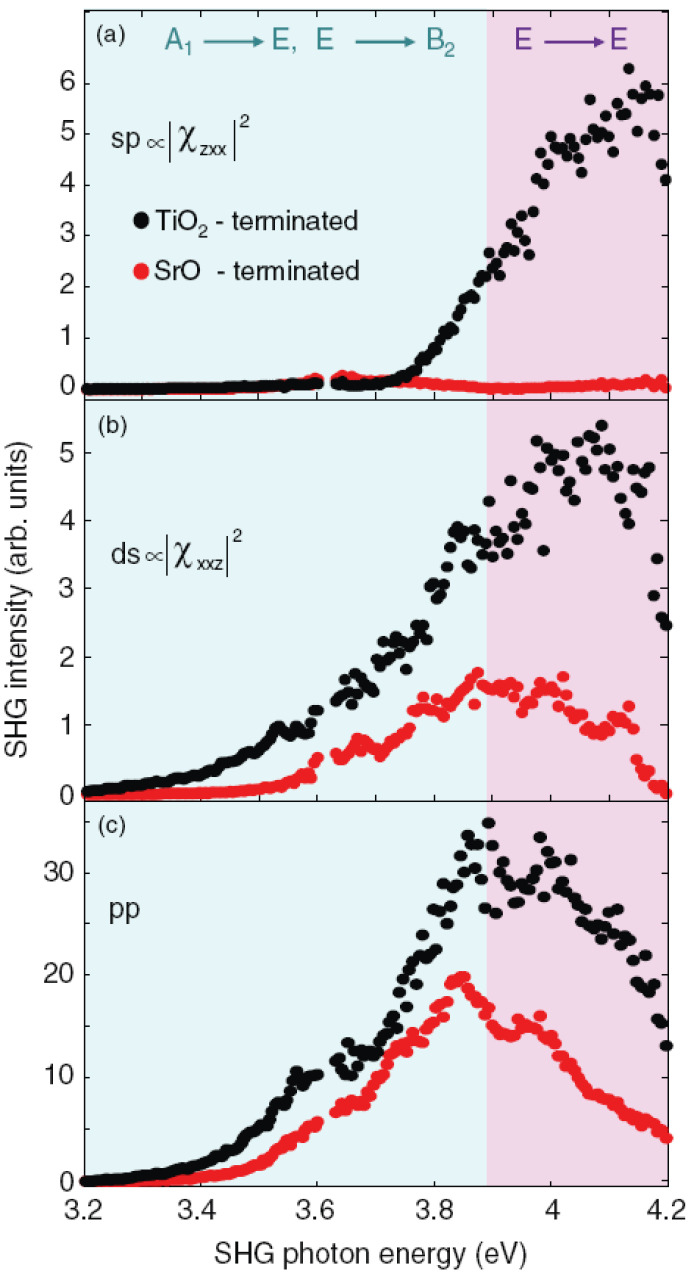
Comparison of SHG spectra from LAO/STO hetero-structures with five LAO unit cells and different interfacial atomic terminations. The labels sp (**a**), ds (**b**), and pp (**c**) refer to different combinations of ω/2ω polarizations (see text). The spectra for the SrO-terminated substrate (red symbols) are multiplied by a factor of 4 for better visibility. The colored areas divide the graph into two energy ranges corresponding to the identified electronic transitions: the blue area is dominated by A${}_{1}\to$ E, E → B${}_{2}$ transitions, while the purple area is driven by E→E transitions. Reprinted with permission from Ref. [39].

**Figure 6:**
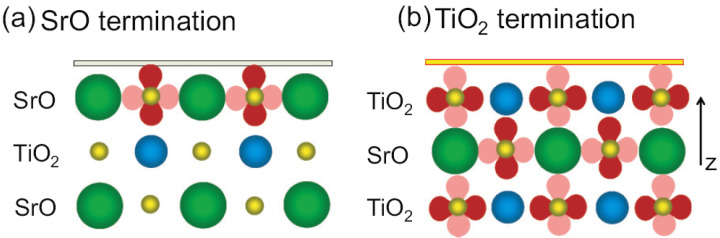
A schematic image of the two possible STO terminations. The 2*p* oxygen orbitals are shown in dark red and pink, where dark red highlights direction maximizing overlap with the Ti atom. In the case of SrO-terminated STO 2$p_{z}$ orbitals point towards the underlying Ti atoms, thus favoring orbital overlap. Reprinted with permission from Ref. [39].

The most important result of the SHG spectroscopy is summarized in Figure 3, where the assignment of each orbital transition to a specific element of the tensor $\chi^{\left(2\right)}$ is shown. The observed transitions are very robust as they are the source of SHG for all studied parameters. The 4mm symmetry was found to be generally preserved, albeit with minor but interesting deviations. Furthermore, the overall enhancement of the low-temperature SHG signal, and consequently an increase in polarity at the interface, supports the picture of a spatial displacement of the titanium ions, which are prone to move at lower temperatures due to the quantum para-electric nature of STO. Slight deviations from this general picture, as dictated by the symmetry selection rules, are (i) the suppression of the 3.6 eV peak in the sp spectra is not always complete, showing that the symmetry is partially lifted due to a distortion of TiO${}_{6}$ octahedra; and (ii) the SHG signal is related to the in-plane lattice mismatch of the LAO/STO, NGO/STO and LGO/STO insulator interfaces, demonstrating that substrate distortions are probably induced or at least modulated by strain in the epitaxial film. These results clearly prove that fine details in the interfacial structure are reflected in, and revealed by, SHG spectroscopy. However, where this detailed sensitivity is most striking is in the study of the influence of substrate termination on LAO/STO electronic properties. The 2D electron gas is actually observed only for the TiO${}_{2}$-terminated STO substrate (*n*-type doping, TiO${}_{2}$/LaO interfaces), while for the SrO-terminated substrate (SrO/AlO${}_{2}$ interfaces) a *p*-type conductive interface is predicted based on the polar catastrophe scenario, but the samples are found to be insulating for any LAO coverage and growth condition [6,7]. SHG Spectroscopy in this case shows a clear qualitative difference between the spectra of TiO${}_{2}$ and SrO terminated samples, as can be observed in Figure 5. The 3.8–4.0 eV peak is strongly suppressed in the sp spectrum of SrO terminated interfaces with respect to TiO${}_{2}$ ones. In Ref. [39], a detailed resonance analysis has been performed and the the model presented in Figure 6 summarizes the resulting findings, as explained in the following.

The almost complete suppression of the 3.8–4.0 eV peak in the sp spectrum is attributed to a suppressed transition E→E: O$(p_{x},p_{y})\to$Ti-$T_{2g}\left(d_{xz},d_{yz}\right)$. Since this cannot be explained by the selection rules, as both terminations share the same interfacial symmetry, it must be assumed that in the SrO interface the SHG is mainly generated in the orbitals located within a single atomic plane from the interface, as these are strongly polarized due to structural proximity effects. Only the oxygen $p_{z}$ orbitals have significant overlap with the Ti(3d) orbitals of the underlying TiO${}_{2}$ layer, so that only transitions involving $p_{z}$ should be pronounced in the spectrum. Thus, the transition E→E: O$(p_{x},p_{y})\to$Ti-$T_{2g}\left(d_{xz},d_{yz}\right)$ of the sp spectrum is suppressed in SrO-terminated systems. Furthermore, the transitions $A_{1}\to E$: O$(p_{z})\to$Ti-$T_{2g}\left(d_{xz},d_{yz}\right)$ remain possible, and contribute to the non vanishing ds and pp spectra. However, here the $E\to B_{2}$: O$(p_{x},p_{y})\to$Ti-$T_{2g}\left(d_{xy}\right)$ transition will be suppressed. Conversely, in TiO${}_{2}$ terminated samples, the charge injection that drives the electronic reconstruction at the interface creates a space charge region spanning a few unit cells, as already mentioned above, thus displacing the Ti ions and developing an electric field that polarizes all the orbitals involved in the SHG emission. Thus, the overall SHG yield will be higher and all symmetry-allowed O(2p)→Ti(3d) transitions will contribute to the SHG spectrum. The suppression of the sp signal in the SrO-terminated samples is strong evidence that only the highest STO atomic plane at the interface is significantly polarized, which, in turn, implies that no significant charge injection occurs in the SrO-terminated samples, unlike the TiO${}_{2}$-terminated samples. These findings have helped ruling out some of the proposed models for explaining the lacking of *p*-type doping in LAO/STO hetero-structures, and, in particular, all those based on charge carrier trapping.

## 6. Non-Standard LAO/STO Hetero-Structures

**Figure 7:**
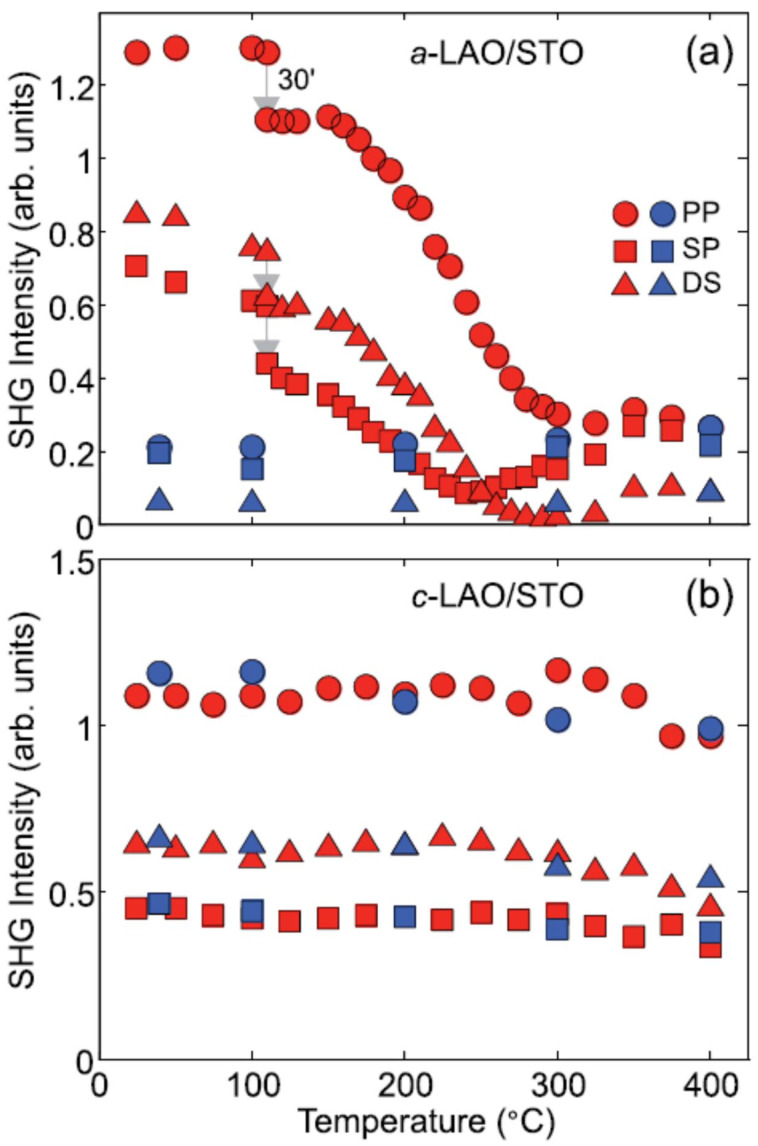
SHG intensity as a function of temperature for 10 equivalent-unit-cell thick *a*- and *c*-LAO/STO samples (panel (**a**,**b**), respectively), for three polarization combinations: pp (circles), ds (triangles), and sp (squares). Red (blue) points refer to the heating (cooling) stage. The arrow at 110 ${}^{\circ }$C indicates the beginning of the SHG signal decrease. At this temperature a pause of about 30 min (30’ label in the figure) is performed before continuing the temperature scan. Reprinted with permission from Ref. [46].

As opposed to the case of different terminations where, according to the polar catastrophe model, the SrO-terminated interface is expected to be conductive and is in fact insulating, there is the case of amorphous-LAO structures in which no polar build-up can be postulated since the polar LAO layer is grown on the STO substrate in an amorphous phase and yet electric conduction at the interface is observed. Additionally, in this case, SHG was found to be capable of helping to address the apparently inconsistent observation [46]. Figure 7 shows a temperature scan of the SHG signal from amorphous-LAO (*a*-LAO, panel a) and crystalline-LAO (*c*-LAO, panel b) upon heating (red datapoints) and cooling (blue datapoints). It is also worth noting that during the measurement, the temperature was held at 110 ${}^{\circ }$C for half an hour, for reasons explained below. It was already shown by transport measurements that, contrary to *c*-LAO structures where conductivity is recovered almost 100% after any number of thermal cycles, in the case of *a*-LAO the 2D electron gas is lost after the very first cycle, indicating an extrinsic origin of the doping charge, and, in particular, pointing to oxygen vacancies, for the simple reason that heating the sample in air has the same effect as filling the oxygen vacancies at the interface, which, in turn, can be formed by ion bombarding during PLD growth of the samples. The SHG results in Figure 7 confirm that the doping mechanisms that induce the interfacial polarity detected by SHG are very different in the two material systems. For the *c*-LAO/STO sample, the behavior of the SHG signal as a function of temperature is qualitatively the same as that observed for the conductivity. The SHG signal is approximately constant during both the heating and cooling phases. After the cooling step, the SHG signal from the *c*-LAO/STO interface recovers approximately its initial value at room temperature. In the case of *a*-LAO/STO, a pronounced decrease in the SHG signal is observed, analogous to the conductivity. However, SHG also exhibits some significant features which are not visible otherwise. The decrease in the SHG signal already begins at about 110 ${}^{\circ }$C: above this temperature, the SHG signal decreases spontaneously at an approximate rate of about 30% per hour. This spontaneous change does not take place for conductivity, which starts to decrease only above 200 ${}^{\circ }$C. After this break, the temperature is increased again by 10 ${}^{\circ }$C every five minutes. In addition, the relative strength of different SHG components are irreversibly changed by heating; the ds signal decreases more rapidly than the sp one, until it vanishes at about 300 ${}^{\circ }$C, while the other two components never become zero. Below about 250 ${}^{\circ }$C, ds is always greater than sp, while this relationship is reversed when above 250 ${}^{\circ }$C. Finally, the SHG signal remains constant at the values reached for the highest temperatures upon cooling. Therefore, the ds signal remains lower than that of sp, thus reversing the initial signal hierarchy of these two polarization combinations. This result shows that the obtained insulating interface is different from the untreated single crystal STO surface since the latter generates an sp signal which is always lower than that of ds, provided that the energy of the SHG photon is not close to the optical resonances of STO. The naive picture of an oxygen-free interface which after heating and subsequent oxygenation becomes identical to the pristine strontium titanate surface is probably true, but at least partially misleading or incomplete. The most remarkable observation is that the different evolution of SHG and electrical conductance under a thermal treatment clearly indicates two different donor mechanisms at the *a*- and *c*-LAO/STO interfaces, oxygen vacancies in the former case and electron reconstruction driven by interfacial polar discontinuity in the second case. The question then arises naturally, why is a similar SHG signal observed for all the *c*- and *a*-LAO/STO hetero-structures?

**Figure 8:**
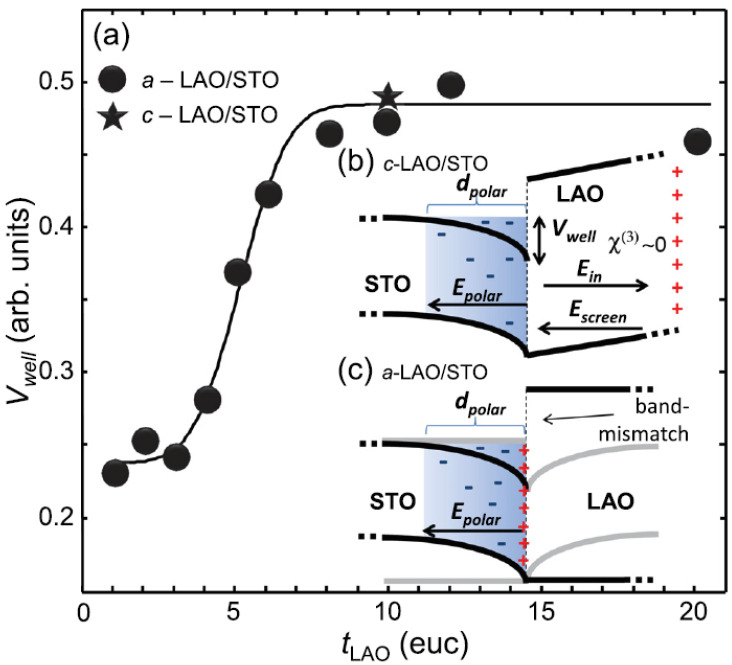
In panel (**a**) the values of the potential-well depth V${}_{well}$ for *a*-LAO/STO (circles) and *c*-LAO/STO (star) extracted from the SHG signal are reported as a function of LAO thickness $t_{LAO}$ (in euc, i.e. equivalent unit-cell). The solid line is just a guide for the eyes. In the insets (**b**,**c**) the possible band diagrams of an *c*-LAO/STO and *a*-LAO/STO interface are shown. For the *c*-LAO/STO interface, the bending of the LAO bands is due to the built-in electric field, $E_{in}$, as predicted by the polar catastrophe model. At the critical thickness, this field is offset by $E_{screen}$. The band structure of the *a*-LAO/STO interface is tentatively represented as a typical potential well derived from a δ-doping. The latter implies that the electronic states on the LAO side (represented by gray wings) are rendered inaccessible by the mismatch of the Fermi level between STO and LAO. The latter is highlighted by the gray lines on the STO side, which show the band structure in the absence of δ-doping. Reprinted with permission from Ref. [46].

As already mentioned, SHG indirectly probes the charges and their spatial distribution at the interface. When a space-charge region is created at the interface, this develops an electric field, $E_{polar}\left(z\right)$, that polarizes the electronic orbitals involved in the SHG process. This contribution may be accounted by the third term of Equation (1), where one field is given by $E_{polar}\left(z\right)$ and the other two are the optical field at ω. Note that the structural contribution to SHG is still described by the second term of Equation (1). By assuming ${\bar{\chi }}^{\left(3\right)}$ slowly varying over the thin layer $d_{polar}$ affected by the electronic reconstruction one finds that

(9) $${\bar{\chi }}_{\mathrm{eff}}\propto {\bar{\chi }}^{\left(2\right)}+{\bar{\chi }}^{\left(3\right)}\int E_{polar}\left(z\right)dz={\bar{\chi }}^{\left(2\right)}+{\bar{\chi }}^{\left(3\right)}V_{well},$$

where the bar indicates the spatial averaging over the *z* coordinate. The variation of ${\bar{\chi }}_{\mathrm{eff}}$ at fixed ${\bar{\chi }}^{\left(2\right)}$ is therefore proportional to the depth $V_{well}$ of the potential well induced by the charge distribution at the interface. An estimate of $V_{well}$ as a function of LAO thickness is provided in Figure 8 for both the *a*-LAO/STO and *c*-LAO/STO interfaces, with an average of the polarization combinations. All crystalline samples above the conductance threshold have an interfacial $V_{well}$ similar to amorphous samples in the saturation region. This result indicates the existence of a universal depth of the interfacial potential well, despite the fundamentally different doping mechanism acting in these two material systems. A possible microscopic scenario for the case of the *a*-LAO/STO interface, which could help explain this similarity, is based on the hypothesis that a δ−doping mechanism caused by oxygen vacancies takes place at the *a*-LAO/STO interface. The band diagram of *a*-LAO/STO would, therefore, be determined by the combination of two basic elements, i.e., the typical “dip” of the bands formed in the vicinity of a δ-doping sheet and the well-known misalignment of the conduction band of LAO and STO, making the states on the LAO side of the δ-doped interface inaccessible to electronic wave functions. The interaction of these two components would lead to a band diagram such as the one in Figure 8c, showing significant similarities, on the STO side, to the one predicted by the electron reconstruction model for the *c*-LAO/STO case, as shown in Figure 8b. One possible explanation is that this depth is actually dominated by the difference between the minimum energy of the conduction band in the STO bulk and the Fermi level, a difference which should be largely independent of the properties of the interface.

**Figure 9:**
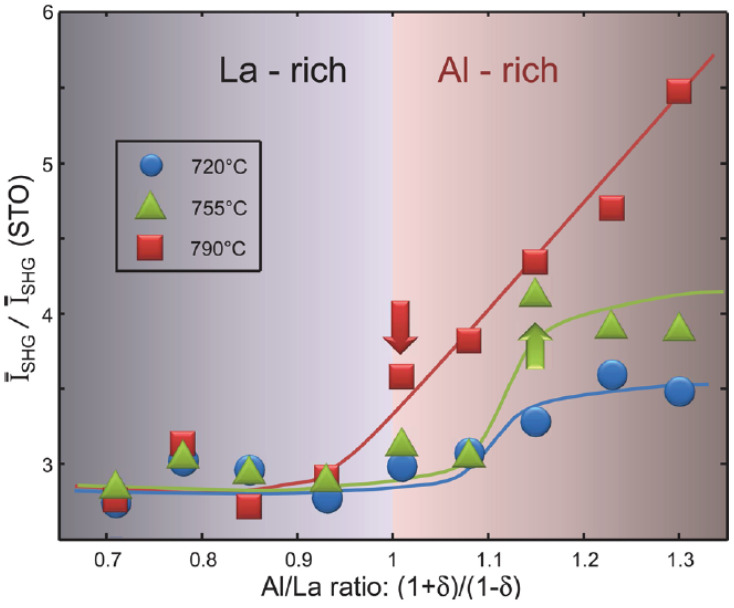
The total yield of SHG as a function of the Al/La ratio, normalized to the value found for the STO–air interface. The two arrows indicate the onset of conduction for the 790 ${}^{\circ }$C-set (red) and the 755 ${}^{\circ }$C-set (green) samples. The shaded areas denote the two non-stoichiometric chemical compositions, respectively, rich in La and Al. The shade gradient shows the direction of increase in the chemical component in each respective area. Solid lines are a guide for the eyes. Reprinted with permission from Ref. [48].

Another interesting case of non-standard LAO/STO hetero-structure is the one with unbalanced chemical composition. In particular, Ref. [47] shows that samples with nominal formula La${}_{\left(1-\delta \right)}$Al${}_{\left(1+\delta \right)}$O${}_{3}$ were grown by molecular beam epitaxy (MBE), and only Al-rich samples were found to be conductive. In Ref. [48], SHG has been deployed to study such interfaces, and the main result is shown in Figure 9, where different batches of samples grown at different temperatures and with different chemical composition are compared. The signal of the samples grown at about 720 ${}^{\circ }$C is only slightly increasing in the Al-rich region of the diagram, while the other two groups show a much more pronounced increase. The sample richer in Al, in particular, shows an increase in SHG of about 50% compared to that of the 755 ${}^{\circ }$C set with the same Al/La ratio, while the conductivity for these samples is approximately the same or even slightly higher for the set grown at 755 ${}^{\circ }$C. The most remarkable thing is that the conductivity of the 720 ${}^{\circ }$C samples saturates by increasing the Al content, but SHG does not demonstrate the same behavior. In contrast, the 755 ${}^{\circ }$C sample set shows similar saturation behavior to the corresponding conductivity. In both cases, the doping level at which SHG starts to increase for the 755 ${}^{\circ }$C and 790 ${}^{\circ }$C set correspond to the start of conduction (indicated by the red and green arrows in Figure 9). The reason for the lack of conduction in the La-rich samples was proposed by first-principles calculations in Ref. [47]; in the La-rich films the excess was not found to substitute on the Al sites of the LAO crystal because it is too large, forming Al${}_{2}$O${}_{3}$-vacancy complexes instead. Conversely, in Al-rich films the Al is incorporated in place of the La sites. This leads to few cation vacancies in Al-rich films vs. many cation vacancies in La-rich films, which results in a significantly higher diffusion coefficient for cations in the La-rich films. In turn, this makes it possible to offset the diverging electric potential via cations migration. On the contrary, in the Al-rich films, the low diffusion coefficient of cations forces an electronic reconstruction in order to compensate the diverging electric potential, thus leading to the formation of a 2D electron gas. Conversely to conductivity, at the higher substrate temperature, the SHG signal does not saturate, since an increase in Al atoms is directly accompanied by the formation of new active SHG defect dipoles. The same behavior is not observed at lower substrate temperatures, probably due to the ability of excess Al to hinder crystallization in the La${}_{\left(1-\delta \right)}$Al${}_{\left(1+\delta \right)}$O${}_{3}$ film. Under Al-rich growth conditions when the growth temperature is low, a large volume of the La${}_{\left(1-\delta \right)}$Al${}_{\left(1+\delta \right)}$O${}_{3}$ film is amorphous and Al excess in this amorphous matrix does not produce defect dipoles. On the other hand, increasing the substrate temperature counteracts this glass-forming tendency. A substrate temperature of about 790 ${}^{\circ }$C is sufficient to maintain the La${}_{\left(1-\delta \right)}$Al${}_{\left(1+\delta \right)}$O${}_{3}$ crystalline film everywhere so that Al substituting onto the La sites may produce defect dipoles. This explains the anomalous behavior of SHG as a function of Al/La ratios for the samples grown at the highest temperature.

**Figure 10:**
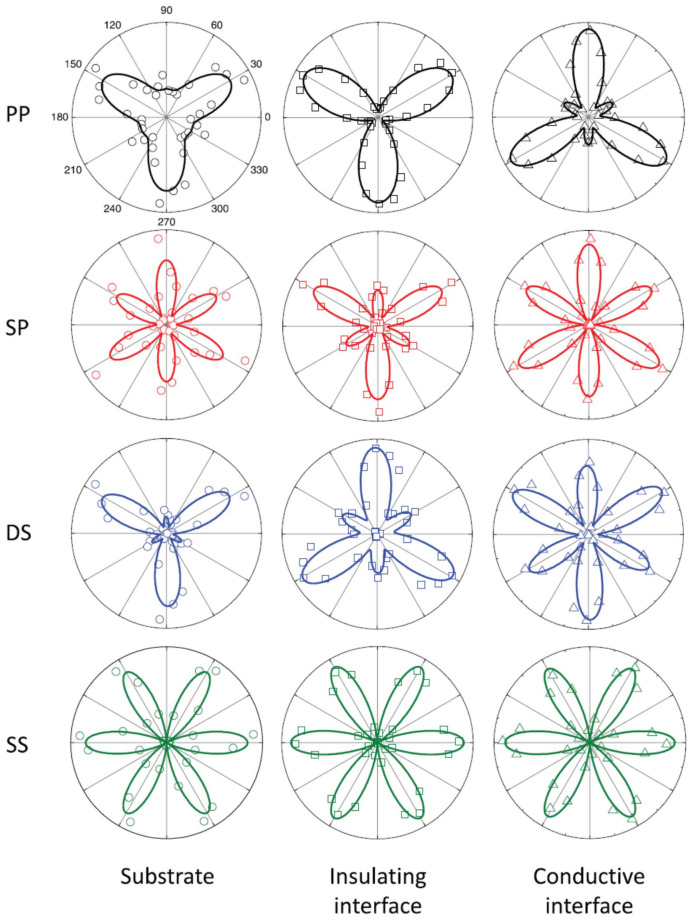
SHG Polarimetry: the signal is shown as a function of the azimuthal angle ϕ and polarization combinations (pp, sp, ds, ss) for LAO/STO(111) samples. Open symbols are data and the solid lines are best-fit curves according to the 3 m symmetry group. Reprinted with permission from Ref. [37].

Another example of the SHG technique applied to non-standard LAO/STO hetero-structures is provided by interfaces with different in-plane orientations, the LAO/STO (110) interface is an interface formed by the deposition of LAO atomic layers on oriented STO substrates (110). According to the polar catastrophe scenario, the LAO/STO interface (110) is formed by a sequence of ${\left[O_{2}\right]}^{4-}{\left[\mathrm{LaAlO}\right]}^{4+}$ planes over ${\left[O_{2}\right]}^{4-}{\left[\mathrm{SrTiO}\right]}^{4+}$ and thus should not be conductive. Conversely, the LAO/STO interface (111) also displays a polar discontinuity, being formed by an alternative sequence of ${\left[\mathrm{Al}\right]}^{3+}{\left[{\mathrm{LaO}}_{3}\right]}^{3-}$ over ${\left[\mathrm{Ti}\right]}^{4+}{\left[{\mathrm{SrO}}_{3}\right]}^{4-}$ planes, and thus should be practically comparable to standard LAO/STO(001) interfaces. In fact, all these interfaces show the same insulator-to-metal transition. At first sight, this result may appear to challenge the polar catastrophe picture. However, recent theoretical studies [49] show that at the LAO/STO (110) interface the STO surface is not an ideal stoichiometric surface, but the ground state is characterized by a buckled TiO termination. This predicted ionic structural distortion leads again to an interfacial polar discontinuity, that, in turn, drives the electronic reconstruction through the same mechanism of the (001) interface. In order to confirm or disprove this particular theoretical prediction, SHG was employed in Ref. [37] as the ideal experimental tool to probe the existence of a ionic interfacial polarity at the (110) interface. Figure 10 shows an example of the SHG Polarimetry performed on LAO/STO (111) samples. A detailed symmetry study on (001), (111), and (110) orientations has demonstrated that the theoretical picture of Ref. [49] is indeed correct, and, in particular, that the interfacial polarity is already very strong before the onset of conduction for the (110) samples, with no appreciable signs of multi-polar contributions, and this comes directly to the surface structural distortion of the STO (110) substrate, which undergoes the formation of a buckled TiO termination.

## 7. SHG Imaging

**Figure 11:**
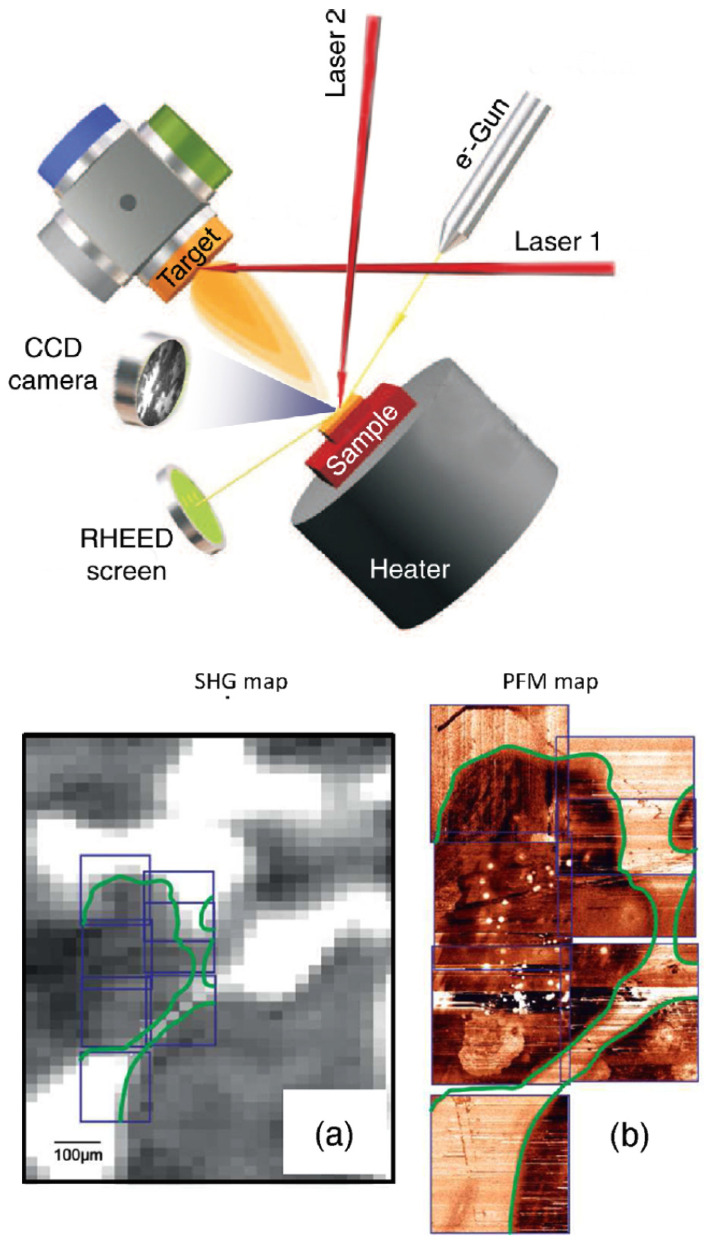
(**Upper Panel**): Schematic layout showing a possible device concept for in situ monitoring of epitaxial film growth by SHG. The usual geometry of the PLD chamber is not affected by the presence of an additional laser beam since both the source and the detector could be located outside the growth chamber. (**Lower panels**): (**a**) SHG intensity map on a SrTiO${}_{3}$/NdGaO${}_{3}$(110) sample and (**b**) PFM image of the same area showing the electrostatic potential measured in contact mode (bright regions correspond to a higher electron density). The colored lines are guides for the eye. Reprinted with permission from Ref. [56].

The possible presence of local inhomogeneities in the electronic structure of the interface was proposed [50,51,52,53] to account for the delayed onset of conductivity at n=4 instead of n=3, as measured with non-transport techniques, such as SHG. In addition to the problem of free/bound charges, magnetic [13], and chemical [54] phase separation has been reported too. SHG was found to be one of the very few techniques able to address these points, because of its ability to investigate the in-plane spatial distribution of the interface reorganization, through the lateral degrees of freedom in the focal plane. SHG can be, in simple words, used to make a “polar picture” of the sample. The resulting picture will not represent the morphological properties of the surface, but rather the strength of the polar nature of the buried interface. In Ref. [55], the SHG Imaging technique has been used in order to address this issue, and quite surprisingly it was found that all hetero-structures with n≠1, including the substrate itself (n=0), are very homogeneous, while only for a single mono-layer of LAO coverage, distinct inhomogeneities are visible, which are characterized by the formation of bright regions with a lateral extension of ≃30 μm. The case of n=1 seems to be quite special. Electronic and chemical inhomogeneities have been found to agree with the observations, although the latter is less plausible, while oxygen vacancies clustering was rejected for its failure to account for the absence of any observable structure at n≥2. In Ref. [56], SHG Imaging has been proposed as a tool for non-invasive, non-destructive, real-time, in situ imaging of oxide epitaxial film growth. Figure 11 shows the proposed idea and the preliminary measurements which are showing the feasibility of the device. In the upper panel, a simplified sketch of a Pulsed Laser Deposition (PLD) setup is shown equipped with the standard Reflection High Energy Electron Diffraction (RHEED) setup and the new proposed SHG imaging tool. The films can be monitored during growth with lateral resolution of ≤1 μm on a very large area (about 1 cm${}^{2}$). The potential of the device is demonstrated by an ex situ analysis of thin epitaxial SrTiO${}_{3}$ films grown on (110) NdGaO${}_{3}$ substrates, taken as prototypical example of a perovskite-based hetero-interface, such as LAO/STO and many others. Of course the real future device should be able to work on a larger variety of materials in order to be fruitfully implemented in real PLD chambers, but it can be taken as a proof of principle. The example picture shown in panel Figure 11a indicates the presence of large regions with pronounced polar nature, which have been independently observed by Piezo-response Force Microscopy (PFM) in order to investigate the electrostatic potential of the sample surface. In addition, a 180${}^{\circ }$ phase shift in SHG occurs when the order parameter of a broken-symmetry is reversed, as happens for instance in the case of adjacent ferroelectric (FE) domains with opposite polarization. The possibility to investigate the SHG signal phase is a relatively easy task which can be obtained by interference between the signal from the target sample and that of a reference sample, and, thus, this makes it possible to distinguish between FE domains having the same size of FE polarization, but reversed orientation. The SHG imaging provides complementary information to the well-established in situ RHEED and it can reveal otherwise elusive in-plane inhomogeneities of electrostatic, chemical, or structural nature. We are not going here to review the entire field of in situ SHG monitoring because it goes beyond the scope of the present review work, but we remind the reader to the comprehensive review on this specific topic in Ref. [57] and references therein.

## 8. Time-Resolved SHG

The last topic we would like to cover in this review is the dynamic behavior of charge carriers in LAO/STO. So far, only the static state at the interface has been discussed, although fast and ultra-fast dynamics would introduce a powerful degree of freedom for understanding the nature of charge carriers. In Ref. [58], a pump-probe scheme is used in which a pump beam with photon energy of 4.35 eV, i.e., above the direct band gap of STO, can excite photo-carriers which, in turn, induce a change in interfacial polarity. The latter is probed by the SHG of a second infrared beam as completed in standard static experiments. The results of these pump-probe experiments show that the polarization state can be optically enhanced or attenuated in picoseconds. A physical model based on the effects of charge propagation at the interface and transient polarization build-up is proposed to explain these observations. Time-dependence allows, in fact, to separate the various interactions that contribute to the interfacial ground state across the different time scales on which they respond to optical excitation. For example, transient absorption spectroscopy shed light on the strong influence of LAO film on self-trapped polarons at the LAO/STO interface [59]. The SHG dynamic study revealed the mechanisms driving the accumulation and depletion of light-induced polarity at the interface: the authors found an induced sub-picosecond optical change of interfacial polarity greater than 50%. This surprising effect is driven by the competition between three different mechanisms, screening, asymmetric drift, and trapping. Taking Equation (9) into consideration again, the dynamic quantity measured in the pump-probe mode can be considered as follows:

(10) $${\bar{\chi }}^{\left(2\right)}\left(t\right)={\bar{\chi }}^{\left(2\right)}+\Delta {\bar{\chi }}^{\left(2\right)}\left(t\right)$$

where *t* indicates the time-delay between the pump and the probe pulses, the bar indicates the spatial averaging over the *z* coordinate. The time-dependence is fully included in the second term of the sum, whose components, according to Equation (9), can be written as:

(11) $$\Delta {\bar{\chi }}_{ijk}\left(t\right)=\Delta V\left(t\right){\bar{\chi }}_{ijkl}^{\left(3\right)}$$

**Figure 12:**
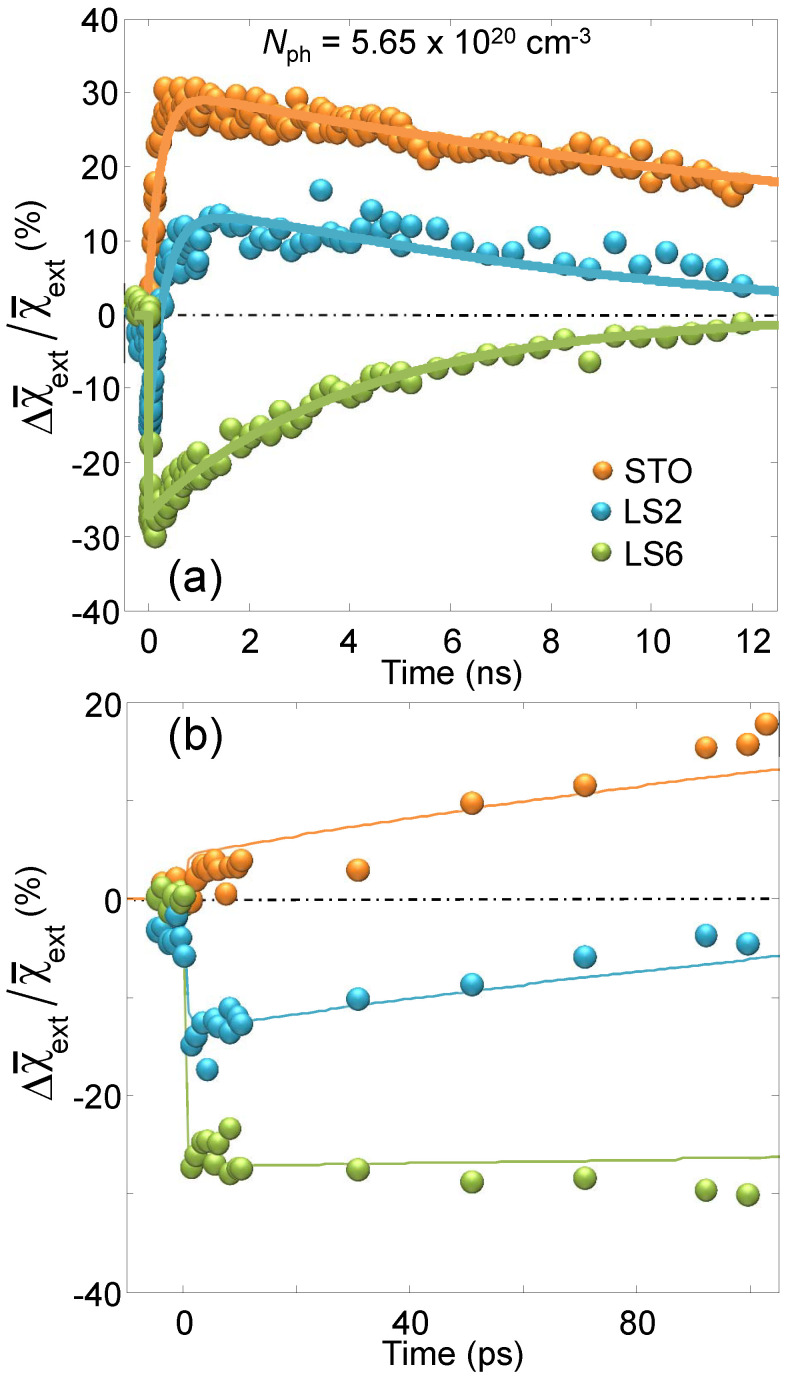
(**a**,**b**) Temporal evolution of $\Delta {\bar{\chi }}_{ext}/{\bar{\chi }}_{ext}$ for a pump photon density $N_{ph}=5.65\times 10^{20}$ cm${}^{-3}$. The label ext indicates the $\chi_{zxx}$ component, while LS stays for LAO/STO and the number indicates the LAO overlayer unit cells. Solid lines are best-fit curves according to the dynamical model proposed. Note the striking difference of the dynamics between insulating (orange and blue points) and conductive (green points) samples. Reprinted with permission from Ref. [58].

**Figure 13:**
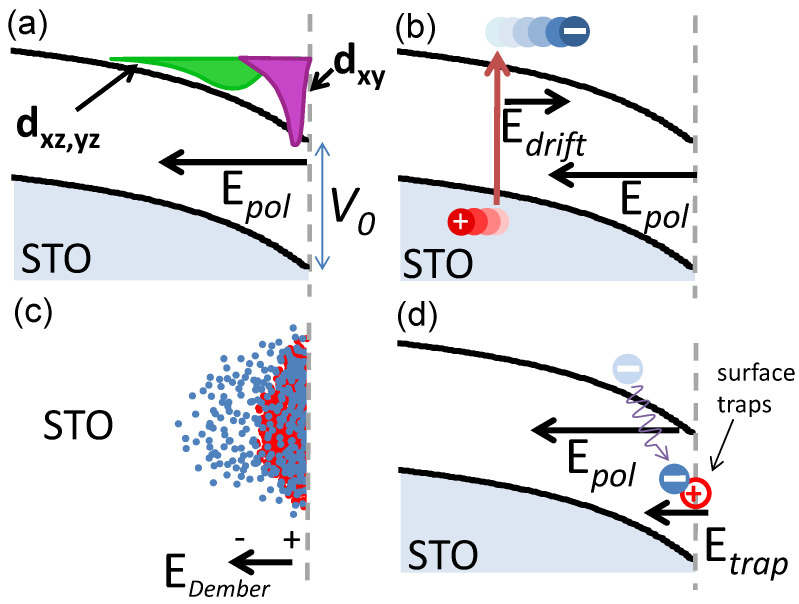
Non-equilibrium charge dynamics near the LAO/STO and air/STO interfaces. (**a**) Band bending and different space depths of charge concentration associated, respectively, with the $d_{xy}$-like and $d_{xz,yz}$-like sub-bands of STO at the interface. We note that the medium beyond the dotted line can be air, as in bare STO, or a LAO overlay. (**b**) Screening drift as the first mechanism inducing the interfacial polarity change. (**c**) Photo-Dember effect as a second mechanism inducing the interfacial polarity variation. (**d**) Interfacial charge trapping as a mechanism inducing a transient polarization. Reprinted with permission from Ref. [58].

In other words, it is assumed that the main dynamical effect is captured by the time evolution of the quantum well energy depth $V\left(t\right)=V_{0}+\Delta V\left(t\right)$ induced by the interfacial electric field. Here, the authors refer to ${\bar{\chi }}_{loc,ext}$ in order to indicate the $\chi_{xxz}$ and $\chi_{zxx}$, respectively. This distinction highlights the more “localized” or “extended” nature of the electronic transitions identified for those two components according to the more pronounced nature of the respective final states ($d_{xy}$ for localized and $d_{xz,yz}$ for extended) [51]. Figure 12a and the zoomed-in view in Figure 12b are showing the dynamical behavior of the “extended” component $\chi_{zxx}$ for two representative samples with n=2 (LS2) and n=6 (LS6) LAO coverage. The SHG signal shows a pronounced and ultrafast drop within less than 1 ps, followed by a quick recovery (on the 100 ps time scale) and a subsequent, and much slower restoration, of the ground state (in several nanoseconds). What is striking and interesting is the fact that both the substrate and the insulating interface (STO and LS2 in the figure) present an “overshooting”, meaning that the polarity becomes larger than that of the ground state after the initial ultrafast drop, so that the recovery to unexcited level of the signal happens in the positive range (polarity decreases to unexcited level), while for the case of conductive sample (LS6) the initial drop is much more pronounced and the recovery happens completely in the negative range (polarity increases to unexcited level). Exploiting a comprehensive investigation about the change in linear reflectivity, LAO coverage, and photo-excitation energy density, a relatively simple model, depicted in the sketches of Figure 13, has been proposed to interpret the complex dynamics of these electron systems. It is seen that the dynamics is the result of several different and competing mechanisms, whose microscopic origins will be roughly explained in the following, with the help of the four sketches of Figure 13. Panel (a) shows the band bending at the interface in the ground state and highlights the more localized nature of the $d_{xy}$ orbitals (purple) compared to the more extended nature of the $d_{xz,yz}$ orbitals (green); panel (b) shows the drift of the photo-carriers in the presence of a pre-existing equilibrium electric field (“screening drift” mechanism), whose effect is mainly captured by the ultrafast drop of the SHG signal within the first picosecond; panel (c) shows the so-called photo-Dember effect, i.e., a transient and local electric field originated by the anisotropic diffusion of electron vs. holes which induces a net shift of the electron cloud with respect to the hole cloud (“charge-propagation” mechanism); finally panel (d) shows the interfacial charge carrier trapping (and subsequent recombination) as the process contributing to the increased interfacial polarity (“transient polarization” mechanism). In order for the latter mechanism to obtain the observed evidence, the enhanced interfacial polarity, it is worth noting that the charge sign of the trapping centers must be positive for inducing an electric field concordant to the existing quantum-well field. It is necessary to point out that this mechanism would work equally well in the case of other kind of charge accumulation, such as, for instance, the formation of large polarons at the interface, although charge trapping is the most likely scenario, in particular because of the well-known presence of oxygen vacancies (positive trap sites). By the study of the fit function parameters derived from this model, and by comparison with literature [60], it was possible to demonstrate that polarons are good candidates to explain the observed transition state. The lattice deformation accompanying the polaron state must break the inversion symmetry to be visible in SHG, as occurs near the interface where the photo-generated polarons are trapped by defects and become polar. The most important evidence to this conclusion can be drawn by the comparison with the model parameters and the known drift mobility of polarons in STO, and this demonstrates how much important the dynamical studies are, even if the ground state, rather than the excited states, is the main target of the investigation.

## 9. Conclusions

In this work, we have reviewed more than 10 years of experiments applying SHG to the investigation of oxide hetero-structures with a particular focus on the textbook LAO/STO interface. The results clearly show that SHG is capable of providing complementary information on these material systems compared to the standard tools that are widely used in material science. The advantages of SHG compared to the latter are many-fold: (i) SHG can reach vertical spatial resolution on the subnanometric scale; (ii) it is a contact-free technique; (iii) it can directly sense interfacial polarity; (iv) it can be used in situ and real-time for monitoring the hetero-structure growth without disturbing the particle trajectories; and (v) it can reach temporal resolution on the femtosecond time-scale.

Let us summarize here what we believe are the main results in the field. SHG spectroscopy revealed a reorganization of the electronic structure at the interface at n=3 which precedes the formation of the 2DEG at n=4. This reorganization is invisible to other techniques which are sensitive to the conductive charges only. Its extreme symmetry breaking sensitivity made it possible to observe directly the spectral influence of the atomic termination of the SrTiO${}_{3}$ substrate and the influence of extrinsic carrier doping, especially at low temperature. Exploiting the lateral spatial degrees of freedom of SHG, the transverse plane homogeneity of the interface is revealed. Lateral inhomogeneities on a length scale of ≈30 μm are found in samples with n=1 epitaxial mono-layer of LaAlO${}_{3}$ (and absent in n>1 samples), due to a phase-separation involving domains with different electronic and chemical properties. Studies on different chemical composition (stoichiometry of the components) and crystalline structure (crystalline vs. amorphous hetero-structures) helped to single out the role of so-called intrinsic effects (polar catastrophe) from the extrinsic effects (oxygen vacancies and others). SHG time-resolved experiments on the femtosecond-to-nanosecond time-scales revealed a stunningly complex carrier dynamics after optical excitation, which can be regarded as the result of a competition between at least three different dynamical relaxation channels. These results shed light on the occupancy and lifetime of electronic states, and suggest that light pulses can be exploited to enhance or attenuate the polarization at the interface and possibly the charge density and/or mobility.

On the other hand, it can be difficult to directly link the SHG signal to the microscopic properties of the interface under study, so that often the interpretation of the SHG results remains confined to a qualitative level. Enhancing the diagnostic and predictive capacities of SHG would require a major effort on the microscopic theoretical modeling of SHG, thus allowing it to compete with other powerful tools in material science, as photo-emission spectroscopy or scanning tunnel microscopy. Notwithstanding these limitations, some of the results here summarized have proved that SHG is able to provide valuable information on the physics of these interfaces that cannot be easily obtained with other techniques. The main strength of SHG is its capacity of sensing all the charges injected at the interface, both localized or mobile. In the case of the STO-based interfaces it is clear that SHG is more sensitive to the first kind of charges, thus allowing to reveal a phenomenon that acts as a precursor for the onset of conductivity. This precursory interfacial reconstruction, due to charges that are injected but localized at the interface, cannot be of course highlighted by the electrical measurements routinely used to characterize these interfaces. This is even more true if we consider LAO/STO interfaces with different terminations of the (100) STO substrate. In this case, there are few techniques that may provide a significant comparison between these two interfaces, and SHG is one of the simplest. The results obtained by SHG spectroscopy in this particular case are simply striking. First, they show that SHG is able to provide useful information on the level of a single atomic layer. Second, they prove that, at the SrO terminated LAO/STO interface, charges are not injected, an important piece of information, not possible to obtain with other techniques.

Moreover, SHG can directly measure the interfacial polarity and even the interfacial electric field that builds-up on charge injection. In this respect, SHG spectroscopy has unequivocally demonstrated that SrTiO${}_{3}$ is a fundamental ingredient for the formation of the 2DEG gas at these interfaces. Injected charges spread in a thin layer on the STO side and there they build-up a polar electric field leading to the formation of a confining quantum-well. SHG can measure directly the depth of this quantum-well, thus allowing, for instance, to single out universal characteristics of fundamentally different systems as amorphous and crystalline LAO/STO interfaces and to call into question once again the fundamental role played by SrTiO${}_{3}$ in the physics of these material systems.

In addition, SHG spectroscopy as a function of temperature has shown that the polar asymmetry induced by the build-up of the polar electric-field is always accompanied by a structural reorganization of the interface due to the displacement of Ti ions that are very much prone to shift from their equilibrium position because of the paraelectric nature of SrTiO${}_{3}$. This is another important piece of the puzzle representing the physics of these material systems, since it has been lengthily debated if the charge injection induces a structural or electronic reconstruction of the interface; SHG results show that both are in place.

In conclusion, this review article has provided several evidences to convince the readers that Second Harmonic Generation may be an invaluable experimental tool for investigating buried interfaces (LAO/STO and other oxide hetero-structures) which share common chemi-physical characteristics. In this conclusive section we have summarized the major results that, in our opinion, have been obtained by means of SHG and which make this technique unique in comparison with other standard tools used in this field. Along with the review, we have delivered several examples highlighting the richness of information that SHG can gather, with the aim of providing a good starting point for further developments in this fruitful research field.
